# Case report: Systemic presentation of ALK-positive Histiocytosis

**DOI:** 10.3389/fonc.2024.1366766

**Published:** 2024-04-19

**Authors:** Yongbao Wei, Ruochen Zhang, Deng Lin, Xiaoyan Chen, Lizhi Li, Haijian Huang

**Affiliations:** ^1^ Shengli Clinical Medical College of Fujian Medical University, Fuzhou, China; ^2^ Department of Urology, Fujian Provincial Hospital, Fuzhou, Fujian, China; ^3^ Department of Pathology, Fujian Provincial Hospital, Fuzhou, China; ^4^ Department of Pediatric Surgery, Fujian Provincial Hospital, Fuzhou, China

**Keywords:** ALK-positive histiocytosis, ALK, male genitourinary system, KIF5B, rare disease

## Abstract

ALK-positive Histiocytosis (ALK-HSs) is a recently identified rare clinical entity characterized by tissue histiocytic alterations associated with ALK gene rearrangement. Clinical presentations can be solitary, multifocal, or systemic (involving multiple sites and organs). Due to limited reported cases, there is inadequate understanding of this disease. This report presents a case of ALK-HSs in a 71-year-old male patient who presented with hematuria for one week. Imaging studies conducted at an external hospital showed multiple lesions in the penis, bilateral testes, back skin, and the third lumbar vertebra. Histopathological findings included spindle and histiocytic cell proliferation with mild or indistinct cellular atypia, interstitial infiltration of lymphocytes, plasma cells, foamy histiocytes, and fibrous tissue proliferation. Immunohistochemistry of the lesion cells revealed positivity for CD68, CD163, ALK1, ALK (D5F3), and Vimentin. FISH testing indicated ALK gene separation in the lesion cells. NGS testing identified the fusion genes KIF5B(NM_004521) and ALK(NM_004304) in the lesion cells. We combined the characteristics of this case with a review of the literature to enhance our understanding of this rare clinical entity.

## Introduction

ALK-positive Histiocytosis (ALK-HSs) is a rare proliferative disease of histiocytes. In the 2022 fifth edition of the “WHO Classification of Tumours of Hematopoietic and Lymphoid Tissues,” this disease type has been added as a provisional subtype (1). In recent years, scholars have gradually recognized and reported ALK-HSs. The prognosis for this condition is primarily favorable, but in a few cases, it can be poor. Searching in the international medical literature, fewer than 50 cases have been reported to date, making systemic ALK-HSs extremely rare (2, 3). This article presents one case of systemic ALK-HSs, detailing the patient’s treatment, follow-up, and prognosis. It summarizes the clinical and pathological characteristics of systemic ALK-HSs and reviews relevant literature to provide a basis for clinical treatment.

## Case report

The patient is a 71-year-old male presenting with hematuria for one week. Physical examination revealed a bladder mass and imaging from an external hospital showed multiple masses in the penis, bilateral testes, and the third lumbar vertebra, ranging from 0.5 to 4 cm in diameter. Upon specialized examination, multiple nodules were found in the bilateral testes, penis, and back skin. These nodules were firm, poorly mobile, solid to touch, non-tender, and had clear boundaries. Imaging studies revealed slightly enlarged bilateral testes with multiple hypoechoic nodules (4 on the left, 3 on the right), the largest measuring 11.3mm x 10.3mm on the right and 10.4mm x 7mm on the left, roughly circular without distinct capsules, and displaying linear blood flow signals internally. Ultrasound contrast indicated mild nodular enhancement, showing quick uptake and slow washout. A hypoechoic area was observed in the right testicular sheath, measuring approximately 51 x 24mm. Additionally, both epididymis appeared normal in size and morphology, with head nodules measuring about 4mm x 4mm, exhibiting clear boundaries and good echogenicity. Color Doppler flow imaging showed normal blood flow in both testes and epididymis without significant abnormalities in the blood flow spectrum.

**Figure 1:**
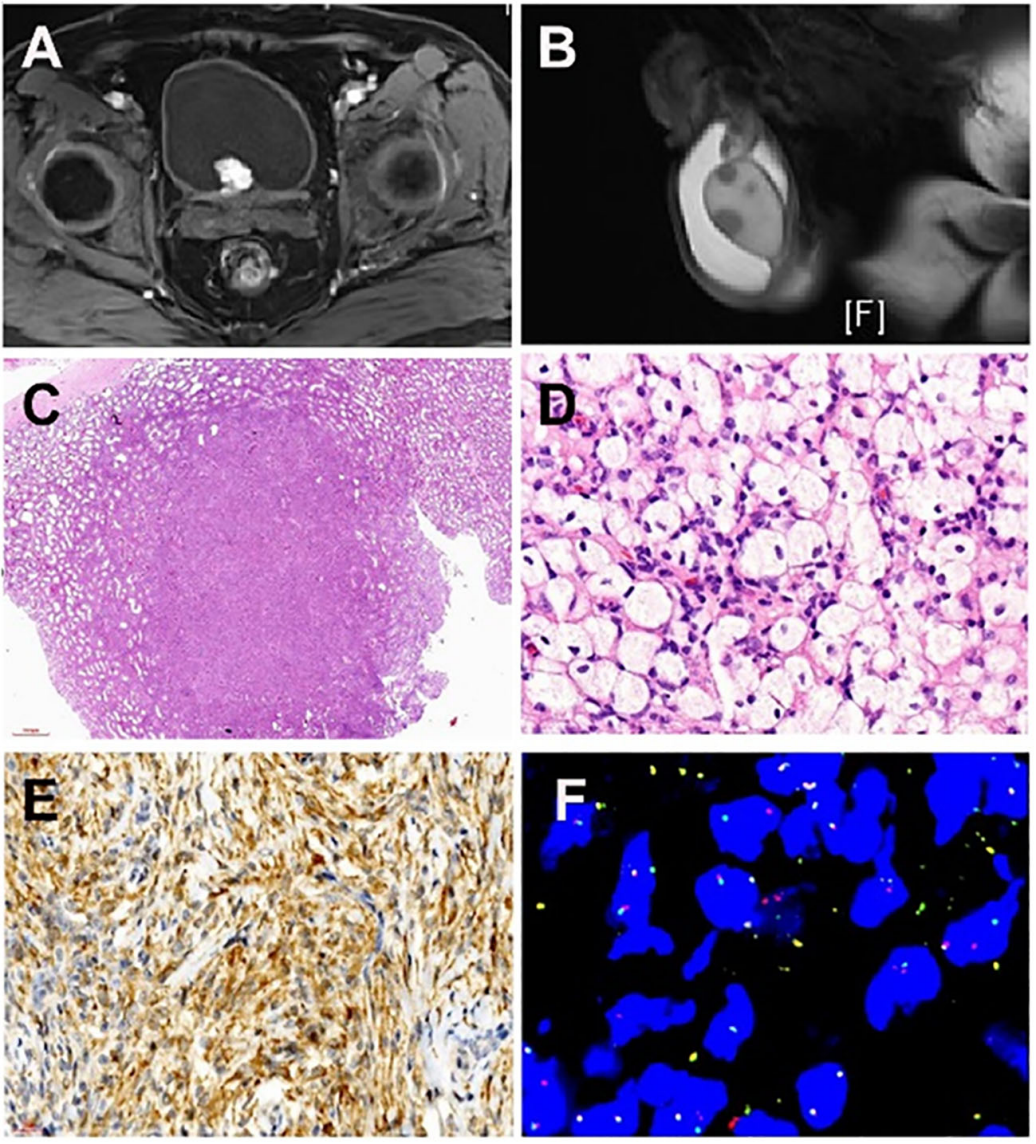
Imaging of a patient with systemic manifestation of ALK-positive Histiocytosis. **(A)** Magnetic resonance imaging (T1-weighted imaging) displays a cauliflower-like soft tissue mass measuring approximately 2.0cm x 2.1cm x 1.9cm, exhibiting clear margins situated at the posterior lower wall of the bladder. **(B)** Magnetic resonance imaging (T2-weighted imaging) illustrates numerous small nodular lesions within both testes, revealing fatty suppression on T2WI and measuring approximately 0.8cm in diameter. **(C)** Multiple nodular distributions lacking capsules are observed under low magnification, scattered within the testicular parenchyma, displaying relatively clear boundaries of the nodules. **(D)** Lesions resemble xanthoma-like structures with the proliferation of foam-like tissue cells characterized by large cell volumes, irregularly folded or lobulated nuclei, and fine nuclear chromatin. **(E)** Immunohistochemical examination of the testicular mass displays positivity for ALK1 (cytoplasmic positivity). **(F)** Fluorescence *In Situ* Hybridization testing was conducted on the testicular and bladder masses, revealing ALK1 gene rearrangement.

MRI examination indicated a cauliflower-like soft tissue mass, about 2.0cm x 2.1cm x 1.9cm in size, with clear margins, located at the bladder’s posterior lower wall (bladder neck) (**Figure 1A**). The mass displayed iso-intensity on T1-weighted imaging, slightly hyperintensity on T2-weighted imaging, high signal on DWI, low signal on ADC, and uneven mild to moderate enhancement post-contrast administration. Multiple small lymph nodes were found in the bilateral inguinal regions. Additionally, numerous small nodular lesions within both testes showed fatty suppression on T2WI, measuring about 0.8cm in diameter, and hydrocele in the bilateral scrotum (**Figure 1B**). The scan also revealed multiple slightly extended and longer T1 and T2 signal lesions in the bilateral femoral upper segments, pelvic bones, and some lumbar-sacral vertebrae, with some lesions showing significant enhancement post-contrast. This suggested a highly vascularized mass at the posterior lower wall of the bladder (bladder neck), indicating bladder cancer, with other tumorous lesions to be further investigated. Multiple lesions in the bilateral femoral upper segments, pelvic bones, and some lumbar-sacral vertebrae were indicative of metastatic tumors awaiting confirmation.

CT examination revealed a nodular lesion protruding into the bladder lumen in the posterior wall (bladder trigone area) measuring approximately 2.1cm x 1.8cm with a CT value of about 35 Hounsfield Units (HU). There was adjacent thickening of the bladder wall, and post-enhancement scanning showed homogenous enhancement of the lesion with a CT value of approximately 75-98 HU.

### Pathological examinations-macroscopic examination

#### Left testis

Testicular tissue measuring 4.7cm × 4.1cm × 2.6cm with intact surface membrane, gray-white. Multiple nodules were palpated on incision, ranging from 0.4cm to 2.3cm in maximum diameter. Nodules appeared solid, gray-white, and gray-yellow on sectioning, with partial clear boundaries and fine texture without accumulated membrane. Epididymal tissue measuring 1.5cm × 1.1cm × 1.1cm with a gray-red and gray-yellow cut surface and a moderate texture. The diameter of the cut end of the spermatic cord was 0.8cm.

#### Right testis

Testicular tissue measuring 4.7cm × 4.3cm × 2.8cm with intact surface membrane, gray-white. Multiple nodules were felt on the incision, with the largest measuring 0.6cm. Nodules appeared similar to the left testis in color, texture, and structure. Epididymal tissue measuring 1.5cm × 1.3cm × 1.2cm with a gray-red and gray-yellow cut surface, moderate texture, and a cut end of the spermatic cord with a diameter of 0.8cm.

#### Bladder

Several small tissue blocks, collectively measuring 1.3cm × 1.2cm × 0.5cm, are gray-white with a moderate texture.

#### Penis

One nodule measuring 2.2cm × 1.2cm × 1cm with a gray-white and gray-yellow cut surface and a moderate texture.

### Pathological Examinations-microscopic Examination

#### Testis

Low-power microscopy revealed multifocal nodular distribution without capsules within the testicular parenchyma. High-power microscopy indicated spindle cell changes within an inflammatory background resembling early juvenile xanthogranuloma or chronic inflammation. Spindle or plump spindle-like cells showed tissue cell-like appearances arranged in bundles, crisscross patterns, and whirls. Small nucleoli were visible with fine chromatin, without significant cellular atypia, rare mitotic figures, and interstitial infiltration of lymphocytes, plasma cells, and histiocytes (**Figures 1C, D**).

#### Bladder

Morphologically similar changes are observed in chronic inflammation, xanthomatous, and granulomatous lesions. Spindle cells and foam-like tissue cell proliferation with enlarged cellular volume, irregularly folded or lobulated nuclei, fine chromatin, small nucleoli (some containing 1-4 nucleoli), rich eosinophilic cytoplasm, occasionally engulfing lymphocytes, standard cells, red blood cells, or hemosiderin. No coagulative necrosis or multinucleated giant cells were observed.

#### Penis

Similar morphology to the testis and bladder, occasionally showing mixed multinucleated giant cells but lacking Touton giant cells.

### Immunohistochemistry

Positive antibodies: CD68, CD163, ALK1 (**Figure 1E**) ALK (D5F3), Vimentin.

Negative antibodies: CK (AE1/AE3), P40, EMA, S-100, SOX-10, SMA, Myogenin, Desmin, CD1a, CD4, CD30, CD3, CD20, CD63, FXIIIa, Ki67 proliferation index (5%-10%).

Negative staining for CD34, STAT6, INI-1, CD99, CD10, SSTR2. Special stains (PAS, PAM) did not reveal any pathogens.

TB gene detection was negative.

Genetic testing using fluorescence *in situ* hybridization (FISH) showed ALK gene separation signals in lesions of the testis, bladder, and penis (**Figure 1F**).

Second-generation gene sequencing (RNA sequencing) revealed fusion genes: KIF5B(NM_004521); ALK(NM_004304) in lesions of the testis, bladder, and penis

The final pathological diagnosis was confirmed as ALK-HSs. Follow-up for 30 months showed no metastasis or recurrence.

## Discussion

ALK-HSs refers to tissue cell lesions associated with ALK gene rearrangement, first reported by Chan JK et al. in 2008 (4). It presents clinically as solitary, multiple, or systemic (involving multiple sites or organs), more commonly affecting individuals of Chinese and Caucasian descent (5). The age of onset varies, ranging from 2 months to 50 years for individuals with solitary lesions, and those with systemic manifestations, it ranges from neonates to 40 years old. About 70% of patients develop symptoms before the age of 2. In reviewing the literature, there’s no significant gender difference in ALK-HSs occurrence. The male-to-female ratio in solitary cases is 3:2, with 2 cases of unspecified gender. In systemic cases, the gender ratio is 5:5. Solitary ALK-HSs lesions can occur in the breast, skin, liver, spleen, appendix, and sinuses. Systemic manifestations involve the skin, lungs, liver, spleen, kidneys, intestines, bones, and central nervous system within the skull (2, 3, 5–7). The clinical manifestations include pallor, hepatosplenomegaly, anemia, and decreased platelets without fever or decreased white blood cells (2, 3, 5–7). The lesions progressively enlarge with well-defined borders and are generally non-painful. Imaging studies help detect masses in multiple locations.

Its diagnosis requires a combination of clinical imaging, pathology, and ancillary tests (including immunohistochemistry and necessary genetic testing). Pathologically, ALK-HSs in the liver, spleen, lymph nodes, and skin tissues present similarly, with infiltrated cells in the hepatic sinusoids. These cells have irregularly folded or lobulated nuclei, fine chromatin, and small nucleoli, occasionally containing 2 to 4 nucleoli. The cytoplasm is rich and eosinophilic, with vacuoles visible; occasionally, they engulf lymphocytes, normal cells, red blood cells, or hemosiderin. The skin resembles early childhood xanthogranuloma or chronic inflammation, mainly composed of non-lipid cells. The lesion cells resemble those found in the liver but occasionally mix with multinucleated giant cells with wreath-like nuclei, lacking Touton giant cell features. Some morphological features mimic xanthoma, chronic inflammation, and granulomatous presentations, showing an inflammatory background, histiocytes, or foam-like tissue cell proliferation. Careful observation of tissue cell features and necessary immunohistochemistry and special staining are needed for diagnosis. Bladder pathology, in this case, shows inflammatory features with numerous foam-like tissue cells, making it prone to misdiagnosis or oversight. Electron microscopy reveals short cell projections, rich cytoplasm containing mitochondria, rough endoplasmic reticulum, ribosomes, lysosomes, and phagolysosomes without Birbeck granules. There is no ultrastructural evidence of metabolic disorders. Immunohistochemistry shows positivity for CD68, CD163, lysozyme, ALK1 (membrane/cytoplasmic), focal expression of CD45RB, low Ki67 (<2%); negativity for CD20, CD3, S100, CD30, CD1a, and Langerin. Genetic testing differs between solitary and systemic presentations. Solitary cases mainly exhibit the ALK1-KIF5B gene fusion mutation, reported in 6 cases out of 6 tested; systemic cases show ALK gene mutations: ALK-TPM3 (1/8), ALK-KIF5B (5/8), and ALK-COLIA2 (1/8) (2, 3, 5–7).

ALK-HSs need to be differentiated from the following tumors. The first one is epithelioid fibrous histiocytoma, where the lesion is within the dermis, with a well-defined tumor border, often presenting as semi-circular or polypoid; tumor cells are often arranged in a nested pattern, with vacuolated nuclei, small eosinophilic nucleoli, rich eosinophilic cytoplasm, and eosinophilia; collagenization can occur in the stroma; a few cases exhibit whirl-like growth patterns; some may display binucleation or multinucleation, nuclear grooves, pseudo-inclusions, or clear cytoplasm, resembling epithelioid endothelial cell tumor-like or melanocytic lesions. Immunohistochemistry reveals tumor cell positivity for CD30, CD68, CD163, ALK1, ALK (D5F3), and EMA; Genetic testing shows ALK gene rearrangement in tumor cells but with different ALK fusion partners, mainly SQSTM1 (52%) and VCL (30%). In contrast, other rare fusion types include DCTN1, ETV6, PPFIBP1, and SPECC1L (8). The second one is juvenile xanthogranuloma, which is common in children and adolescents, and often occurs in the skin. It has characteristic xanthoma histiocytes, foam histiocytes and Touton giant cells, and can also be mixed with other types of cells, including epithelioid cells, oval cells, spindle cells and eosinophilic histiocytes. The cells have no polymorphism and nuclear division. There are different numbers of lymphocytes, eosinophils, plasma cells and neutrophils in the background. Immunohistochemistry: histiocytes are positive for CD68, CD163, CD4, CD14, factor XIIIa and fasin, negative for CD1a and Langerin, and mostly negative for ALK. The third is Langerhans cell histiocytosis, characterized by infiltration of spindle and histiocyte-like cells, nodular or patchy distribution, eosinophilic or pale-stained cytoplasm, rich cytoplasm, visible nuclear grooves, and one to multiple small nucleoli. The stroma often presents with eosinophil infiltration, possibly accompanied by lymphocyte and plasma cell infiltration. Immunohistochemistry shows positivity for S100, CD1a, and Langerin in the tumor cells; genetic testing reveals BRAF gene mutations in more than 50% cases. The fourth one is Rosai-Dorfman disease. Pathologically, the lesions exhibit alternating areas of cellular sparse and dense regions. In the cellular sparse areas, scattered, larger, lightly stained histiocytes with abundant cytoplasm containing phagocytized material and lymphocytes showing ‘emperipolesis.’ The cellular dense areas display infiltrations of inflammatory cells such as lymphocytes and plasma cells. Immunohistochemistry reveals CD68, CD163, lysozyme, and CyclinD1 expression in the tumor cells. The fifth one is Erdheim-Chester disease, which mainly occurs in bones and occasionally outside bones. Pathologically, the predominant feature is infiltration of histiocytes, exhibiting large cell volumes with foamy cytoplasm containing ‘lipid-like’ substances. The cell nuclei show condensation and many lack engulfed lymphocytes. Multinucleated giant cells may be present, with a lymphocyte and plasma cell infiltration background. Immunohistochemistry reveals expression of CD68, CD163, and BRAF gene mutations detected in 54% to 100% of cases (9). The sixth one is an inflammatory myofibroblastic tumor in the thoracic and abdominal cavities and soft tissues, formerly known as an inflammatory pseudotumor. Pathologically, it shows spindle cell tumor-like proliferation in an inflammatory background, primarily composed of myofibroblasts and fibroblasts. Immunohistochemistry reveals expression of myogenic markers in the spindle cells, such as Actin, SMA, Caldesmon, and Desmin, and approximately half of the cases show detectable ALK protein expression and gene rearrangement. Other differential diagnoses include chronic inflammation, vascular-type fibrous histiocytoma, ALK-positive large B-cell lymphoma, and ALK-positive anaplastic large-cell lymphoma.

Regarding its treatment and prognosis, limited reported cases and research data are available for ALK-HSs. Published articles have mentioned various treatment methods, including watchful waiting, surgical excision, chemotherapy (using dexamethasone and etoposide), and ALK-targeted therapy. Multiple or systemic presentations often require chemotherapy. Monitoring blood counts and liver-spleen condition during treatment is essential, and normalization of liver-spleen size suggests positive treatment outcomes. Prognosis is generally better for multiple lesions, while systemic presentations vary depending on the affected sites. Reviewing literature cases with follow-ups ranging from 2 to 14 years, one case resulted in death, one survived the disease, and the rest showed no abnormalities or disease recurrence (2, 3, 5–7). Recently, some scholars have successively reported isolated cases, raising whether they are related to systemic ALK-HSs, which requires further investigation (10).

## Conclusions

ALK-HSs belong to rare proliferative disorders of tissue cells, with systemic ALK-HSs presenting involvement in multiple systems. Solitary ALK-HSs generally have a favorable prognosis, and some may regress spontaneously. However, systemic ALK-HSs progress rapidly, with some cases having a poorer prognosis. Clinical management necessitates appropriate treatment strategies such as surgery combined with chemotherapy and ALK inhibitors for targeted therapy. Basically, multiple ALK-HSs and systemic ALK-HSs are two clinically and prognostically different conditions.

## Data availability statement

The original contributions presented in the study are included in the article/supplementary material. Further inquiries can be directed to the corresponding author.

## Ethics statement

The studies involving humans were approved by Ethics Committee of Fujian Provincial Hospital. The studies were conducted in accordance with the local legislation and institutional requirements. The participants provided their written informed consent to participate in this study. Written informed consent was obtained from the individual(s) for the publication of any potentially identifiable images or data included in this article.

## Author contributions

YW: Conceptualization, Supervision, Writing – original draft, Writing – review & editing. DL: Data curation, Investigation, Writing – review & editing. RZ: Data curation, Formal analysis, Investigation, Writing – review & editing. XC: Formal analysis, Validation, Writing – review & editing. LL: Data curation, Formal analysis, Writing – review & editing. HH: Conceptualization, Investigation, Writing – review & editing.
